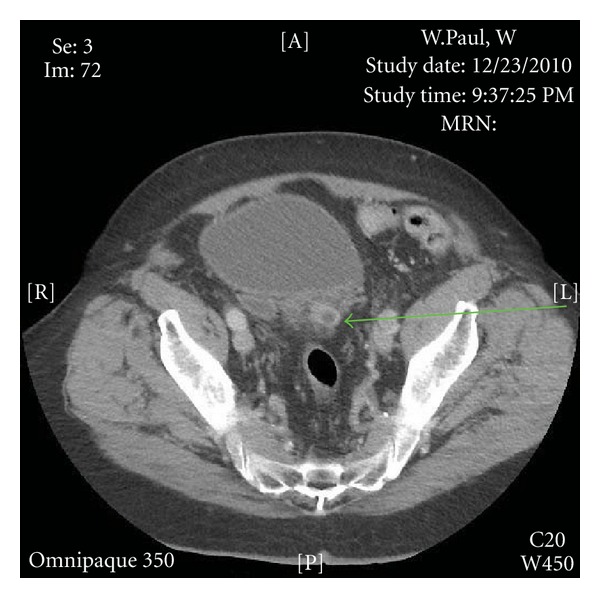# Bladder Diverticulitis: A Case Report

**DOI:** 10.1155/2011/303498

**Published:** 2011-09-22

**Authors:** Michael Silberman, Rebecca Jeanmonod

**Affiliations:** St. Luke's Hospital & Health Network, 801 Ostrum St, Bethlehem, PA 18055, USA

## Abstract

Bladder diverticulum, an outpouching of the mucosa through the muscular wall of the bladder, is a multifactorial disease process that can be either acquired or congenital. Although small diverticuli are usually asymptomatic, a large diverticulum may result in hematuria, urinary tract infection, acute abdomen due to its rupture, acute urinary retention, or neoplasm formation. We describe the case of an elderly gentleman who presented to the emergency department with abdominal pain and was ultimately diagnosed with bladder diverticulitis, a disease not previously described in the literature.

## 1. Case Presentation

An 81-year-old male presented to the emergency department (ED) with bilateral lower quadrant abdominal pain for two-day duration. The patient noted that the pain was band like in distribution, moving entirely from one side of his abdomen to the other, and had been coming and going in 15–20 minute intervals for the previous two days. Several hours prior to admission, the pain began to change and was now a persistent, dull, nonradiating left lower quadrant pain. This changing character of pain made the patient nervous and prompted him to call 911. Associated with the pain, he had an episode of diaphoresis. The patient noted that he had a regular bowel movement earlier in the day. There were no exacerbating or alleviating factors. His review of symptoms was otherwise negative. The patient had a past medical history of a previous urinary tract infection for which he was treated as an outpatient with a course of cephalexin that was completed 7 days ago. Additionally, the patient had a history of atrial fibrillation, hypertension, and benign prostatic hypertrophy. His medication list included cardizem, olmesartan, metoprolol, and a baby aspirin daily. 

Physical exam revealed a stoic gentleman in moderate distress from his abdominal pain, although the patient was appearing to be nontoxic. The patient's abdomen was soft and was tender to palpation in the suprapubic region. There was no rebound, guarding, or costovertebral angle tenderness. There were no appreciable masses. The patient's cardiac, pulmonary, vascular, and skin exams were all normal. The patient was noted to be in rate-controlled atrial fibrillation. 

Given the patient's description of increasing pain along with a history of atrial fibrillation without anticoagulation, a computed tomography (CT) of the abdomen and pelvis with IV contrast was ordered to look for possible mesenteric ischemia. Additionally, a complete blood count, comprehensive metabolic panel, lactate, and urinalysis with culture and sensitivity were ordered. All blood laboratory values were found to be within normal limits. The patient's urinalysis showed small blood and trace ketones. 

The patient's CT of the abdomen and pelvis revealed multiple posterior bladder diverticuli with stranding on the left and enhancement of a left-sided diverticulum consistent with acute bladder diverticulitis (Figure 1). Additional CT findings included a mid-left ureteral calculus with mild left hydronephrosis and multiple nonobstructing bilateral renal calculi. 

The patient was subsequently admitted to the hospital under the medical service for IV antibiotics and pain control, with a Urology Consult. The patient was scheduled to be taken to the operating room the following day for cystoscopy and stone removal. However, the patient felt better in the morning and refused further treatment. He left the hospital against medical advice with a prescription for a five-day course of levofloxacin.

## 2. Discussion

A diverticulum is an outpouching of the lining or the entire wall of any hollow organ in the body [1, 2].As a general rule, regardless of organ involved, these outpouchings can either be congenital or acquired [3]. 

The congenital form of bladder diverticuli usually manifests in children younger than ten years old and is thought to be due to weakness of the ureterovesical junction or a posterior urethral valve [3]. In the former instance, weakness of the junction allows outpouching to occur, and, in the latter instance, increased intravesical pressure leads to wall stress and mucosal outpouching.

The acquired form generally occurs in males over the age of 60, and diverticuli are often located along the lateral bladder walls [3, 4]. Similar to the mechanism in children with posterior urethral valves, it is thought that intravesical pressure increases from other underlying pathology, such as prostatic disease or neurologic processes. An increase in the intravesical pressure causes the urinary bladder mucosa to insinuate itself between muscle bundles, resulting in development of a mucosal extravasational sac or saccule which further results in formation of diverticuli [1, 2]. 

Bladder diverticuli are usually asymptomatic and found incidentally while the patient is being evaluated for an unrelated complaint [5]. However, the larger the size of the diverticulum, the more likely it is for symptoms to be present [5]. Large diverticuli may present with symptoms including hematuria, urinary tract infection, urinary retention, neoplasm formation, or even acute abdomen due to rupture [6]. 

Although diverticulitis in a urinary bladder diverticulum has not been previously described in the literature, the process through which a diverticulum becomes inflamed is likely similar in process to its colonic counterpart, primarily via increased intraluminal pressure or inspissated particles leading to micro- and macroperforation with subsequent inflammation of the diverticulum [7]. Although bladder diverticuli are not uncommon, it is likely that diverticulitis in the bladder has not been described because of the typically sterile environment and lack of solid material in the urinary tract. Simply put, the physiology of the urinary tract makes diverticulitis exceedingly unlikely. 

Although no treatment for urinary bladder diverticulitis has been described, there are several approaches to treat bladder diverticuli [8]. Correction of bladder outlet obstruction is the first line of therapy in patients with diverticuli formed secondary to obstruction, including those with prostatic disease and children with urethral valves [9, 10]. Many of these diverticuli will resolve spontaneously with relief of the obstruction [9, 10]. Should relieving the obstruction fail, or in the event no obstruction is present, a surgical approach with transurethral resection, fulguration, or diverticulectomy is warranted [5, 6, 11]. 

In this particular patient, it was presumed that the patient had diverticuli formation from prostatic disease and that inflammation was likely secondary to one of his numerous renal tract calculi. With no prior cases by which to guide therapy available, the choice was made to treat the disease process as a complicated urinary tract infection. It was hoped that cystoscopy would be performed to obtain a better view of the inflamed area and direct future interventions, but the patient's symptomatic improvement led to his leaving without completion of care.

## 3. Conclusion

Emergency physicians are well versed in the diagnosis and treatment of colon diverticulum and diverticulitis. The same underlying pathophysiology of diverticulum formation and inflammation of the outpouching leading to diverticulitis can be readily applied to any hollow organ. In this case, the hollow organ in question happened to be the bladder, which to this author's knowledge is the first such description. Unfortunately, there is no true proof that a calculus was present within the diverticulum. Although the CT has the appearance of inflammation consistent with diverticulitis, in the absence of confirmatory cystoscopy this diagnosis cannot truly be made with certainty. This patient was treated conservatively with antibiotics alone, and although no further followup has been achieved, the patient has not returned to the emergency department seeking further care.

## Figures and Tables

**Figure 1:**